# The 2025 Expanded Programme on Immunization (EPI) Managers Meeting in West Africa: A Health Systems Analysis of a Decade of Stagnating Routine Immunization Performance

**DOI:** 10.3390/vaccines14060501

**Published:** 2026-06-02

**Authors:** Ado Mpia Bwaka, Marcellin Mengouo Nimpa, Rija Andriamihantanirina, Alain Komi Ahawo, Daman Keita, Evanilda Santos, Desmond Maada Kangbai, Milse William Nzingou Mouhembe, Yves Medessi Armand Mongbo, Tene-Alima Essoh, Christian Tague, Criss Koba Mjumbe, Akpaka Kalu, Benido Impouma

**Affiliations:** 1 World Health Organization, Regional Office for Africa, Brazzaville P.O. Box 242, Congo; bwakaa@who.int (A.M.B.); milse.nzingoumou@who.int (M.W.N.M.); kalua@who.int (A.K.); impoumab@who.int (B.I.); 2 United Nations Children’s Fund, Centre of Excellence for Child Survival and Development, Nairobi P.O. Box 40326, Kenya; 3 Gavi, The Vaccine Alliance, Chemin du Pommier 40, Le Grand-Saconnex, 1218 Geneva, Switzerland; 4 Essential Immunization Programme, Ministry of Health, Conakry P.O. Box 585, Guinea; 5 Essential Immunization Programme, Ministry of Health, Praia 7600-001, Cape Verde; 6 Essential Immunization Programme, Ministry of Health and Sanitation, Freetown P.O. Box 529, Sierra Leone; desmakay@yahoo.com; 7 West African Health Organization (WAHO), Bobo-Dioulasso P.O. Box 153, Burkina Faso; 8 Agence de Médecine Préventive (AMP), Bureau Régional pour l’Afrique, Abidjan P.O. Box 3960, Côte d’Ivoire; 9 Faculty of Medicine, Université Libre des Pays des Grands Lacs, Kyeshero Lusaka Rue 218, Goma P.O. Box 243, Democratic Republic of the Congo; 10 Medical Research Circle, Goma P.O. Box 73, Democratic Republic of the Congo; 11 Public Health Department, Faculty of Medicine, University of Lubumbashi, Lubumbashi P.O. Box 243, Democratic Republic of the Congo

**Keywords:** immunization, West Africa, health system, equity, BCU, vaccine introduction, outbreaks, financing

## Abstract

**Background**: The 2025 EPI Managers’ Meeting for West African countries in Guinea was a critical platform for EPI managers to make an in-depth analysis of immunization programmes. We present a structured analysis of immunization status in West Africa using a WHO Health System model to move beyond descriptive reporting toward systemic analysis for actionable solutions. **Methods**: The meeting convened EPI managers from 14 of the 17 West African countries and partners supporting the immunization program. Country and regional presentations, immunization and surveillance data and meeting discussions were analysed through a framework identifying (1) core problems, (2) systemic barriers using WHO health systems building blocks and (3) actionable recommendations or call for action. **Results**: Analysis revealed stagnating immunization coverage. Recovery from COVID-19 pandemic disruptions remained limited, with persistent outbreaks of vaccine-preventable diseases (VPD). Among the five Immunization Agenda 2030 objectives assessed, only Maternal and Neonatal Tetanus (MNT) elimination was on track. Four critical challenges emerged: (1) Routine immunization stagnation with DTP3 median coverage of 76%. This was associated with challenges related to poor data quality, weak implementation of innovative vaccination strategies and donor dependency, as 88.2% of countries financed less than 50% of routine vaccine costs domestically. (2) Sub-optimal progress in Big Catch-Up (BCU) implementation in some countries, revealing poor health system resilience. (3) Inability to sustain high coverage for new vaccine introductions despite significant progress, highlighting demand and service delivery gaps. (4) Persistent VPD outbreaks with geographical expansion and the resurgence of diphtheria epidemics since 2023. **Conclusions**: Persistent immunization challenges in West Africa appear to reflect interconnected systemic challenges, suggesting the need for a fundamental shift toward subnational strategies, integration of immunization services within primary health care (PHC) and improved data quality. Sustainable financing of the national EPI and acceleration of local vaccine manufacturing is essential to achieve immunization sovereignty in West Africa. Country Call for Action provides strategic guidance to reverse the trend toward the Immunization Agenda 2030 targets.

## 1. Introduction

Like other countries in WHO regions, West African countries are committed to achieving the Immunization Agenda 2030 (IA2030) target of 90% coverage for all vaccines at the national and district levels [1]. The sub-region bears 40% of the WHO African region’s population, with disproportionate immunization vulnerability, with Nigeria alone accounting for 31% of Africa’s zero-dose children, the highest in the region [2].

The annual Expanded Programme on Immunization (EPI) managers’ meetings serve as crucial platforms for assessing progress, sharing innovations, and building consensus [3]. These meetings often involve diverse groups of stakeholders, including Ministry of Health officials, WHO, UNICEF, Gavi and other immunization partners. They play a critical role in aligning stakeholders’ priorities, strategies, and resource allocations. They also enable agreement on priority areas of focus to strengthen and maintain high EPI performance, which gives indications on health systems and primary health care performance.

Traditional EPI managers’ meeting reports often remain descriptive. They catalogue challenges, but do not systematically link to the root causes and solutions [4]. The 2025 West Africa EPI managers’ meeting held in Conakry (Guinea) involved multiple methodological approaches and brainstorming sessions in small groups. The meeting revealed challenges that have led to the stagnation of EPI performance over the last decade.

Moving beyond conventional descriptive reporting, this conference report uses a WHO Health System Framework lens to analyse EPI performances. By applying this model to four critical immunization programme areas, namely routine immunization, BCU initiative, new vaccine introduction, and vaccine-preventable disease (VPD) outbreaks, this report aims to provide a replicable framework that bridges the gap between immunization programmes and health system strengthening, aiming to accelerate progress towards IA2030 goals.

While annual EPI managers’ meetings generate rich descriptive insights and country level discussions, their outputs are often reported without a structured analytical framework linking observed performance gaps to underlying health system determinants. This limits their utility in informing strategic and systemic corrective actions. To address this gap, this conference report applies a WHO health system framework to synthesise meeting outputs.

This study is a conference report-based analytical synthesis with secondary analysis integrating multiple data sources to provide a comprehensive overview of immunization system performance. It is mainly descriptive and does not aim to establish causal inference.

## 2. Meeting Design and Data Collection

### 2.1. Setting and Participants

The meeting convened from 24 to 27 November 2025 in Conakry (Guinea), with EPI managers from 14 of the 17 West African countries (Benin, Burkina Faso, Côte d’Ivoire, Gambia, Ghana, Guinea, Guinea-Bissau, Liberia, Mali, Niger, Nigeria, Senegal, Sierra Leone, Togo) and partners supporting the immunization programme. A total of 221 participants attended in person, and an average of 509 were online. These included national EPI managers, other officers from Ministries of Health, and staff from the WHO, UNICEF, Gavi, WAHO, PATH, AMP, GaneshAID and other immunization partners.

### 2.2. Meeting Format/Methodology

The meeting was organised in four technical sessions in addition to the opening and closing ceremonies. The sessions focused on three main thematic areas: EPI system strengthening (EPISS), vaccine introduction (VCINT), and outbreak preparedness and response (OUTBR). The meeting combined multiple approaches, including presentations from countries and partners, panel discussions, poster exhibitions, and brainstorming sessions in small groups. This enabled a comprehensive and in-depth review of critical problems, root cause analysis, country engagements, and a call for action.

### 2.3. Data Collection and Analysis

Country presentations, synthesis reports from technical working sessions, and the final report of the meeting were reviewed as primary sources of data. These sources were triangulated with national immunization and surveillance databases available at IST WA up to August 2025. WHO/UNICEF Estimates of National Immunization Coverage (WUENIC) and Joint Reporting Form (JRF) 2024 and financing data from countries JRF were also used for this analysis.

The analytical process started by extracting content and organizing it thematically around the core agenda pillars (EPI system strengthening, vaccine introduction, and outbreak control), mapped against the WHO health system building blocks.

Qualitative insights were generated through the structured organization of expert consultation insights during the EPI managers’ meeting. For each agenda pillar and data source, information was systematically extracted and organised into three categories: (1) core problems observed, (2) systemic barriers identified, and (3) corresponding programmatic actions or recommendations. This was based on expert synthesis rather than formal qualitative methods (e.g., thematic coding or reproducibility protocols) and should therefore be interpreted as an analytical and interpretive process.

Quantitative analyses were primarily descriptive and used routine immunization and VPD surveillance data. Analysis included percentages, proportions, and temporal trends. Findings were triangulated with secondary data, including WUENIC 2024 estimates, JRF financing data, and WHO outbreak surveillance reports. Administrative coverage data were compared descriptively with WHO/UNICEF Estimates of National Immunization Coverage (WUENIC) for the same country–year observations. No inferential statistical testing was performed, as the two data sources are based on different methodologies. The analyses were primarily descriptive and did not include formal weighting using multivariate modelling.

### 2.4. Validation and Analytical Rigour

To enhance analytical rigour and reduce interpretative bias, preliminary analytical summaries were triangulated across multiple data sources. Triangulation involved the comparison and synthesis of multiple data sources, including meeting reports, administrative data from countries, and WHO/UNICEF estimates. In addition, a structured expert consultation process involving seven immunization experts from partner organizations and three national EPI managers was conducted to validate the synthesised challenges, health system diagnoses, and proposed strategic actions. Expert feedback was used to refine analytical outputs, ensure accurate representation of the meeting consensus, and support the logical derivation of implications grounded in the West African immunization landscape. In cases where discrepancies were observed between data sources, findings were interpreted through iterative comparison and discussion during expert consultations. Final interpretations were based on convergence across multiple data sources and expert consensus. The inclusion of both programme managers and technical partners further ensured the triangulation of perspectives and minimised the risk of bias.

### 2.5. Conceptual Framework

The WHO health system building blocks framework was used to interpret EPI manager meeting findings and diagnose underlying causes across six domains. These include service delivery, health financing, leadership and governance, health workforce, health information, and medical products and technologies.

**Figure 1:**
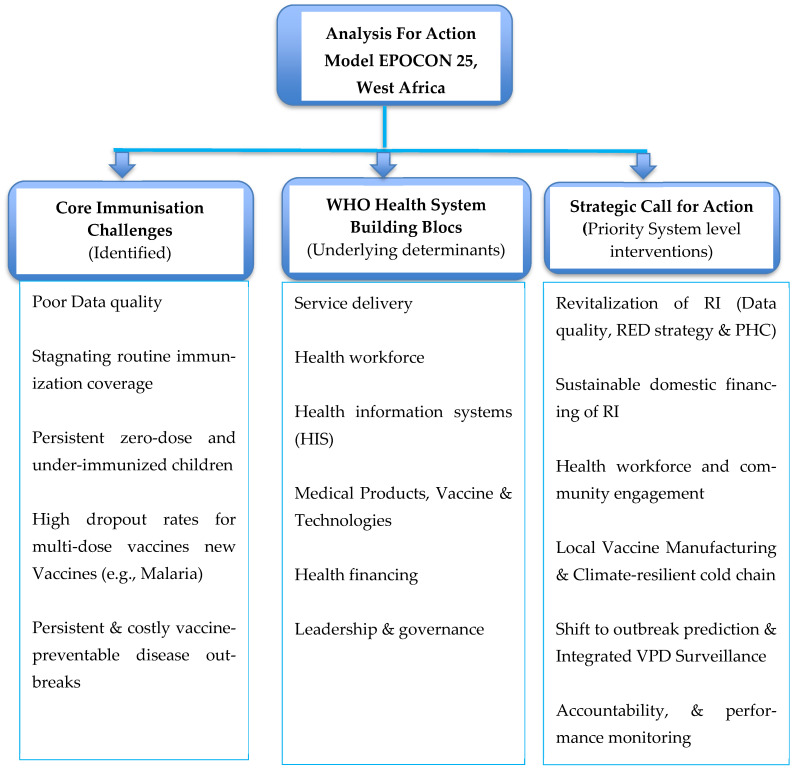
Analysis-for-Action conceptual framework applied to the EPICON25 review.

Building on this framework, a three-step “Analysis-for-Action” model was developed to structure findings by first defining core problems, then analysing systemic barriers using the six health systems building blocks, and finally formulating actionable strategic calls for action grounded in the identified determinants (Figure 1).

### 2.6. Operational Definitions

The following operational definitions were used in this article.

*Zero-dose children:* Children who have not received the first dose of diphtheria–tetanus–pertussis (DTP1)-containing vaccine by 12 months of age [5,6].

*Under-immunised children:* Children who have not received a third dose of diphtheria-tetanus pertussis containing vaccine (DTP3) [5].

*Dropout rate:* Percentage difference between coverage of first and subsequent doses of the same vaccine series (e.g., DTP1 to DTP3) [6].

### 2.7. Ethical Considerations

This analysis used aggregate data from reports (Meeting report, WUENIC, JRF) and publicly available databases. No individual-level data was analysed. The study used secondary aggregated data and did not need an ethics committee review.

## 3. Conference Thematic Developments

### 3.1. Routine Immunization Trends and Data Consistency

**Figure 2:**
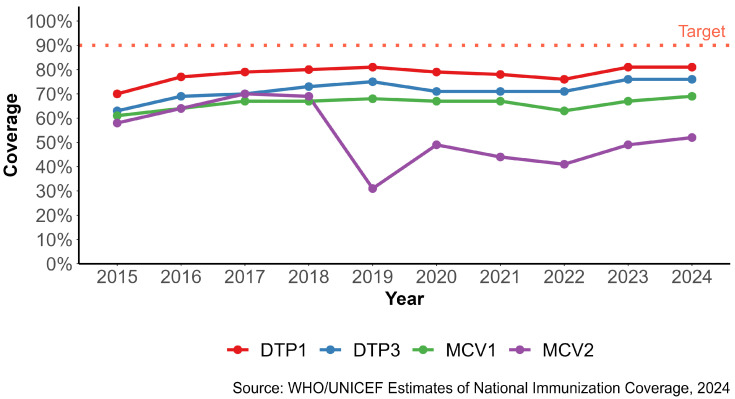
Trends of vaccine coverage for main vaccines in West Africa using WHO/UNICEF WUENIC estimates 2015–2024. Source: Data were obtained from WHO/UNICEF Yearly Estimates of National Immunization Coverage (WUENIC) [2]. Country-level coverage values were used to provide regional trends over time.

Figure 2 summarises trends in DTP and Measles Containing Vaccine (MCV) coverage in West Africa between 2015 and 2024 using WHO/UNICEF estimates of national immunization coverage. Analysis of DTP 3 coverage trends shows a flat trajectory for West African countries over the last decade. The sub-regional median DTP3 stagnated at 76%, below the recommended 90% target. This hides subnational disparities, with 98 of 1479 districts (6.6%) reporting administrative coverage below 50%. This represents an 84% increase compared to the previous year, indicating persistent inequity.

Coverage for the second dose of measles vaccines (MCV2) declined in 2019 following its introduction, with the data particularly influenced by Nigeria, which has the largest birth cohort in the subregion. Across the subregion, MCV2 remained below 60% in IST West Africa.

Data used in Table 1 comes from WHO/UNICEF yearly Estimates of National Immunization Coverage (WUENIC) for all the countries. These were derived from national administrative coverage and national surveys following a standardised WHO and UNICEF methodology. Immunization coverage was categorized as follows: ≥90% (IA2030 target), dark green (optimal); 80–89%, light green (good); 50–79%, yellow (suboptimal); and <50%, red (poor).

Country-level analysis based on WUENIC shows limited progress towards the 90% coverage target. Only Algeria achieved 90% coverage for DTP3, MCV1 and MCV2. No country reached the 90% objective for all antigens simultaneously, based on WUENIC.

**Figure 3:**
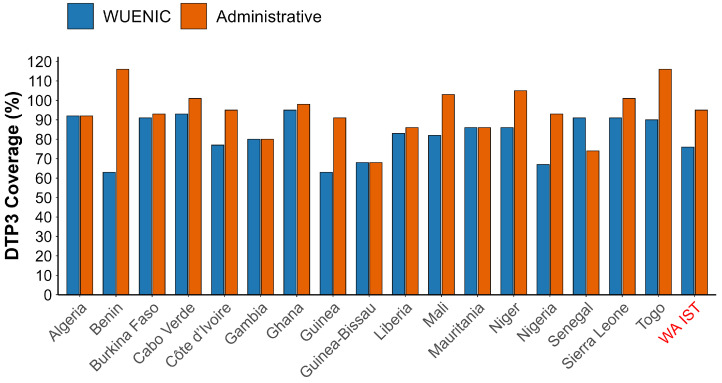
Comparison between administrative and WUENIC for DTP3 in West African countries in 2024 (source: countries’ electronic Joint Reporting Framework (eJRF) and WUENIC 2024) [2,7].

A comparison between administrative coverage data and WUENIC estimates per country (Figure 3) shows the highest administrative coverage, highlighting discrepancies between data sources in most countries (Figure 3). Thirteen countries (76.4%) showed measurable discrepancies between the two data sources, with the largest difference in Benin (53 points). Four countries, Algeria, The Gambia, Mauritania, and Guinea-Bissau, showed minimal or no discrepancy, indicating greater consistency between data sources. The highest discrepancies were observed in nine countries (Benin, Côte d’Ivoire, Guinea, Mali, Niger, Nigeria, Senegal, Sierra Leone, Togo).

**Figure 4:**
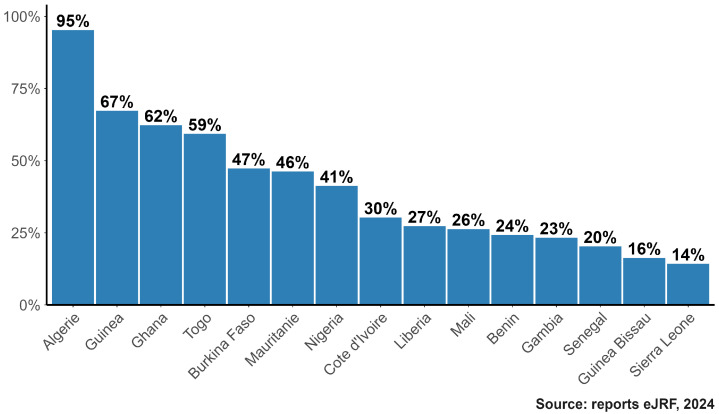
Immunization financing in West African countries in 2024 (source: eJRF 2024) [7].

Analysis of 2024 electronic Joint Reporting Form (eJRF) country-reported data showed that 88.2% countries were financing less than 50% of routine vaccine costs domestically. This shows the dependency of EPI programmes on external donor financing. Only Algeria financed 95% of the immunization programme cost, followed by Guinea, Ghana, and Togo, with the government contributing to 50% to 67% of the immunization programme cost (Figure 4). In nine out of the fifteen countries with data available, over 75% of vaccination expenditure goes to vaccine procurement.

### 3.2. Big Catch-Up (BCU) Implementation Outcomes in West Africa

**Figure 5:**
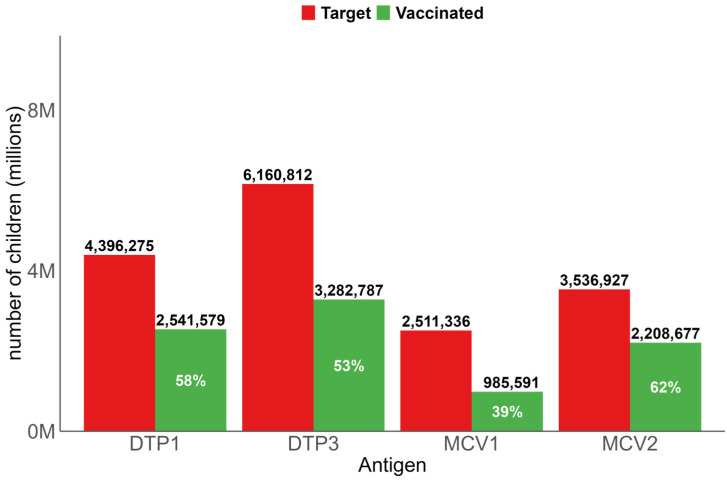
Global zero dose and under-immunised children immunised in IST West Africa. Source: Countries’ administrative data shared with the WHO, aggregated to provide a regional trend [8,9].

Figure 5 presents the number and percentage of zero-dose and under-immunised children reached through the BCU Initiative in West African countries as of September 2025. By that date, the BCU initiative, launched in 2023 to address pandemic-related backsliding, had reached 58% of zero-dose and 53% of under-immunised children. The subregion’s recovery from the COVID-19 pandemic remains incomplete.

**Figure 6:**
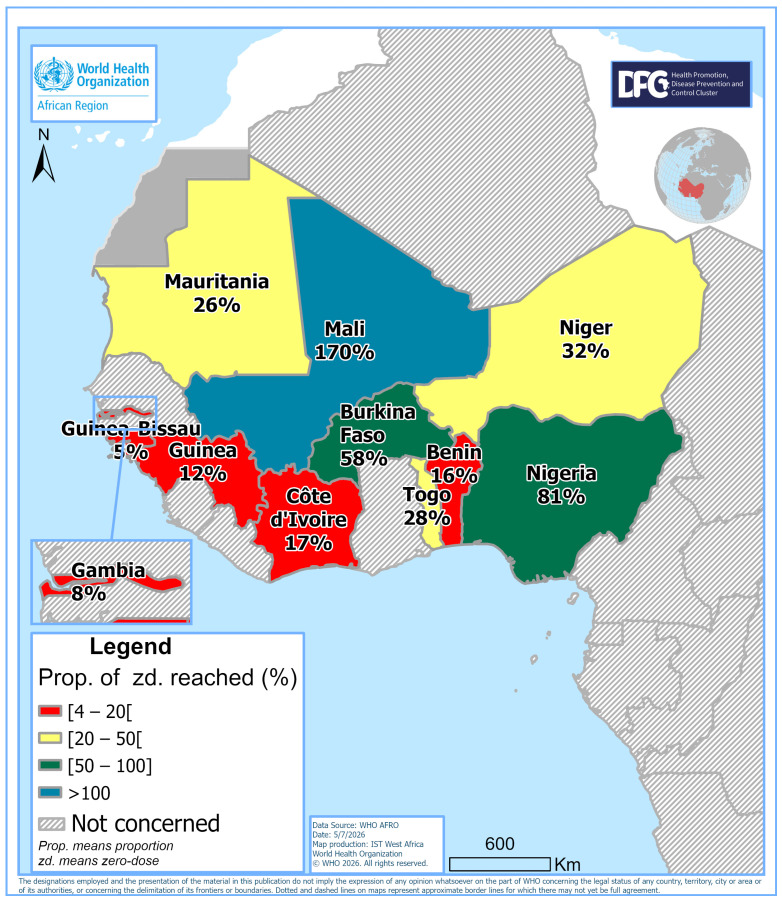
Percentage of zero-dose children reached per country as of September 2025. Source: Countries’ administrative data shared with the WHO compared to expected numbers [8,9].

Different levels of performance were observed across the countries, as shown in Figure 6. While Mali, Niger and Nigeria reached more than 50% of ZD, Benin, Côte d’Ivoire, Gambia, Guinea, and Guinea-Bissau registered low performance, with less than 20% ZD reached.

### 3.3. New Vaccine Introduction Progress

**Figure 7:**
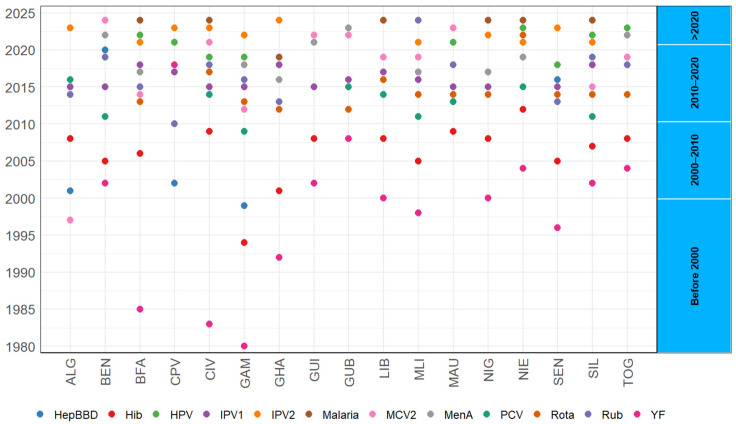
Trends of new and underutilised vaccine introduction in West Africa, 1980–2025. Source: Country yearly reporting on immunization through eJRF data [7]. Data were compiled in a table to show a regional trend.

Figure 7 presents trends in the introduction of new and underutilised vaccines in West African countries between 1980 and 2024. West Africa has made remarkable progress in new vaccine introductions. All the countries introduced at least one vaccine from 2020 to 2024, with a maximum of four in Nigeria, followed by Burkina Faso, Cote d’Ivoire, and Sierra Leone with three vaccines in the last 5 years. New vaccines introduced include malaria vaccine, Human Papillomavirus (HPV), and Typhoid Conjugate Vaccine (TCV) with new targets and delivery strategies.

By October 2025, 11 (65%) out of 17 countries had introduced the malaria vaccine. The hexavalent vaccine has been introduced in Algeria, Mauritania, and Senegal. However, the Hepatitis B birth dose, HPV, and DTP boosters require further rollout, with reemerging VPD outbreaks like diphtheria and pertussis affecting predominantly older age groups.

Early lifecycle data showed a consistent pattern of satisfactory first-dose coverages followed by high drop-out rates. This is mainly seen with multidose schedule vaccines. This trend was observed across many countries, and across newly introduced multidose schedule vaccines. This highlights challenges in achieving sustainable vaccine uptake beyond initial contacts.

### 3.4. Vaccine-Preventable Disease Outbreaks

**Figure 8:**
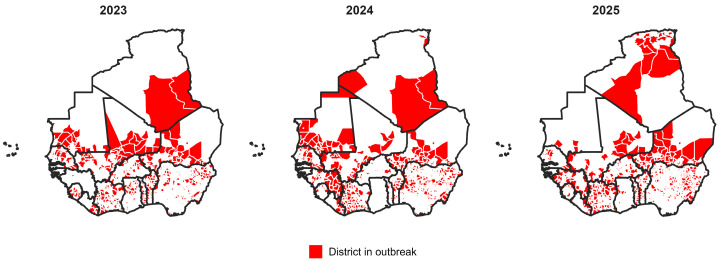
Districts with measles outbreaks in West African countries, 2023 to 2025. Source: Country surveillance databases on measles shared with the WHO [9]. Data were compiled and presented in a map to monitor the geographical expansion.

Figure 8, below, shows a persistent measles outbreak in West African countries between 2023 and 2025 (Week 35). West Africa faces a paradoxical situation in which persistent and expanding outbreaks contrast with regular supplementary immunization activities (measles, polio, yellow fever, diphtheria, etc.). The number of districts with measles outbreaks increased from 337 in 2023 to 452 by week 25 of 2025. This is a 34% spatial increase. During the same period, surveillance performance at subnational levels remained sub-optimal, with 19% of districts failing to meet the two main acute flaccid paralysis (AFP) surveillance indicators [9].

In parallel, a surge in other VPD is observed. West African countries accounted for over 80% of diphtheria cases and of 95% diphtheria-related deaths in the AFRO region [10]. There were 32 confirmed yellow fever cases in six countries in 2025. These patterns occurred despite repeated supplementary immunization activities (SIAs), clearly indicating regional vulnerabilities and raising concerns about the quality and effectiveness of these [11].

## 4. IA2030 Progress Assessment

The immunization programme progress review in West Africa, based on IA 2030 targets, shows that the subregion is globally off track, as summarised in Table 2. Only one of five IA2030 targets is on track, while others are off track or uncertain. Critical systemic gaps that have hindered EPI programmes’ performance over the past decade also present a unique opportunity to address root causes and accelerate progress towards IA2030 targets in West Africa.

## 5. Discussion

The results presented above describe persistent stagnation in routine immunization coverage, widening subnational disparities, and recurrent outbreaks across West Africa. This section interprets these findings through a health-systems lens, examining the systemic underlying constraints and their implications for action. The WHO Health System Framework was used as a conceptual and interpretive lens to structure the findings, rather than as an empirically tested analytical model. Accordingly, the associations discussed reflect an expert-informed synthesis of the evidence and should not be interpreted as evidence of statistical or causal relationships.

### 5.1. Persistent Routine Immunization Coverage Stagnation Hampered by Inaccurate Data and Donor Dependency

The persistent stagnation of routine immunization coverage observed over the past decade suggests system-level constraints across multiple health system building blocks rather than short-term implementation failures. While sub-regional coverage appears stable, the growing number of districts with DTP3 coverage below 50% reveals subnational inequities and sustained pockets of zero-dose and under-vaccinated children (WHO, 2020).

A primary constraint relates to Health Information System Weaknesses, particularly data quality and use. Despite the remarkable deployment of digital solutions for data transfer using DHIS2, many countries still use population estimates from national censuses from more than 10 years ago and paper-based data collection tools at the frontline level, which may affect the accuracy of reported data and influence appropriate decision-making [12,13]. Evidence from a global scoping review on immunization data quality in low- and middle-income countries by Harrison and Wetherill [14,15] revealed that poor-quality immunization data is a persistent problem that undermines accurate assessment of coverage, equity monitoring, and programme decision-making, Data accuracy issues deserve the proper attention of EPI managers, since it may have implications on overall programme management, decision making and effective resource allocation.

Health Financing and Governance is a second major barrier. Evidence from eJRF 2024 [7] suggest a high-level reliance on donor dependency, with 73.4% countries with data financing less than 50% of routine vaccine costs from domestic resources. This high reliance on external financing may be a concern within the current economic landscape, marked by reduced GAVI funding allocation to countries in the next Gavi.6.0 [7,16]. Constraints within service delivery and the health workforce further contribute to coverage stagnation. Immunization service delivery remains fragmented and non-optimal in countries with poor access (below 80%) and poor service utilization rates (above 10%). Immunization programmes’ inability to sustain coverage for multidose antigens suggest sub-optimal demand generation activities with limited use of evidence for Behavioural and Social Drivers Studies (BeSD) for tailored communication [17]. Flourishing immunization-strengthening initiatives (BCU, Zero-dose, etc.) have progressively distanced focus from the effective implementation of the Reach Every Districts (RED) strategy, which remains of paramount importance.

Fragile humanitarian and security contexts may act as amplifiers of these systemic weaknesses. Conflict, population displacements and disruption of primary health care infrastructure exacerbate different constraints, reinforcing the cycle of under-performance and inequity, as documented in other analyses [2].

Taken together, these findings suggest that persistent coverage stagnation in West Africa is best understood as a manifestation of interacting systemic constraints, underscoring the need for integrated, health-system-wide responses rather than isolated technical fixes [18].

### 5.2. Big Catch-Up Initiative Uncover Health System Gaps

The heterogeneous performance of the BCU initiative across West Africa suggests that post-pandemic recovery of immunization services has been primarily shaped by underlying health system strength, rather than intervention alone. Wide variations between countries also demonstrate that weak health system infrastructures reduce countries’ abilities to ensure continuity of care, consistent with other authors’ findings [19].

A major limiting factor relates to non-optimal service delivery capacity. Effective catch-up requires integrated outreach strategies that are embedded within primary health care platforms and responsive to broader community needs. Reaching zero-dose children requires a clear understanding of the main reasons for non-vaccination, which most of the time go beyond vaccination alone [20]. Integrating immunization services within primary health care to address other community needs was not sufficiently addressed by BCU when it was implemented as a time-bound campaign. This should be institutionalised and integrated to ensure sustainable immunization gains [19].

Leadership and governance weaknesses further constrain BCU implementation. Delays in revising immunization schedules in many countries, distributing adapted data collection tools and extending immunization services across life, revealed weaknesses in adaptive management and decentralised decision-making which delayed the kick-off in many countries [21].

The Health Workforce emerged as an additional structural bottleneck. Inadequate and insufficiently motivated human resources for outreach services, coupled with competing priorities, including outbreak response activities (Polio, Measles, YF, etc.) and new vaccine introductions, created pressure on the existing workforce [22]. The poor community health worker network, which varies across countries, also affects programmes’ abilities to identify and enrol zero-dose children within the communities.

Overall, the BCU initiative tested countries’ health system resilience, suggesting that recovery investments may translate into equitable outcomes only where core system building blocks are sufficiently strong [19].

### 5.3. New Vaccine Introduction: The Supply–Demand Challenge

The rapid expansion of new vaccine introductions in West Africa represents a major programmatic achievement. However, a high dropout rate suggests a persistent imbalance between service delivery and demand generation.

A key barrier relates to leadership and governance in vaccine introduction planning. Introduction plans largely prioritised allocating resources to cold chain expansion, logistics, and healthcare worker training, while demand generation remains poorly funded or funding is delayed. This pattern is documented in low-income settings [23]. In the context of growing vaccine hesitancy, timely and evidence-based communication activities should remain key in the introduction of new vaccines.

Service delivery barriers further contribute to a high dropout rate. New vaccines are frequently introduced through vertical campaigns or dedicated sessions rather than being integrated into routine immunization contacts, increasing caregiver burden and system inefficiency, as documented in evaluations of HPV vaccine introduction in Senegal [24]. The limited alignment of new vaccine delivery with existing primary health care platforms undermines long-term uptake.

Together, these findings suggest limited health system capacity to ensure immunization service continuity beyond the second year of life and across the lifecycle. This trend, observed over many countries, suggests the need to strengthen demand-generation activities and improve service delivery and community trust so that the target groups can continue to make the best possible use of vaccination services.

### 5.4. Surveillance Fragmentation and Persistent Outbreaks Requiring Programmatic Shift

VPD outbreak persistence and spatial expansion in West Africa could be associated with three critical failures. These include EPI programmes’ inability to achieve and sustain high levels of routine immunization, sub-optimal supplementary immunization quality, and poor surveillance systems.

A primary constraint lies in the fragmentation of VPD surveillance systems and poor surveillance system performance at subnational levels. Satisfactory surveillance performance at the national level hides significant gaps at subnational levels and delays outbreak detection, investigation and timely response. In addition, VPD surveillance operates in silos. Moving from disease-specific surveillance to integrated VPD surveillance is therefore critical to improving early detection and outbreak prevention under resource constraints, including declining polio funding.

Financing and governance further shape outbreak vulnerability. The VPD surveillance system has been dependent on polio funding in many countries. Reduction and suspension of polio funding, in most countries, is a threat which requires adaptive changes.

The continued reliance on reactive supplementary immunization activities has also reinforced a vicious cycle. Poor routine coverage leads to outbreaks and triggers emergency Supplementary Immunization Activities (SIAs). These responses drain financial and human resources, further disrupting already fragile routine services, creating conditions for subsequent outbreaks. Countries have been working in a reactive mode. This cycle suggests the need for a strategic shift from reactive mode to more proactive and preventive models focusing on risk reduction for targeted, routine immunization-strengthening interventions.

## 6. Limitations

This analysis has limitations. First, it relies primarily on meeting documentation and country reports, which may be influenced by reporting variability across countries and meeting reporters. Second, the study does not include primary data collection or causal inference and uses the WHO Health System blocks mainly for interpreting meeting findings. Third, as a descriptive and interpretive analysis, this study does not include formal measures of uncertainty (e.g., confidence intervals or standard deviations). Findings should therefore be interpreted as programmatic insights derived from multiple data sources and expert synthesis, rather than statistically quantified estimates. Fourth, while the proposed recommendations are evidence-informed, they require contextual adaptation to account for national differences in health system capacity, epidemiology, and governance environments. Fifth, the evolving security situation in some countries may constrain the feasibility and timing of the implementation of certain recommended actions. Finally, although the analysis focuses on four critical programmatic areas, other important topics, including vaccine safety, vaccine hesitancy, and gender-related barriers, were beyond the scope of this study and warrant a dedicated analysis. However, the use of multiple data sources and a structured analytical framework strengthen the validity and usefulness of the findings for strategic planning.

Future studies could consider applying the WHO Health System Framework prospectively, from study design through reporting. The use of formal qualitative methods, including systematic coding of findings and structured prioritisation of recommendations and calls for action, could further strengthen analytical rigour and provide additional insights.

## 7. Strategic Call to Action

The 2025 EPI Managers’ Meeting in West Africa marked a critical juncture, revealing that continuous investment in fragmented immunization and surveillance systems will not deliver the Immunization Agenda 2030 goals. The evidence is unambiguous: stagnant routine immunization coverage, persistent outbreaks, and unsustainable financing demand a fundamental paradigm shift. Based on the analysis, West African EPI managers in Conakry issued a clear call to action for governments, partners, and immunization programmes across the sub-region.

To translate this call to action into implementable guidance, the proposed strategic actions were prioritised according to time horizon and primary responsibility. Actions were organised into short-term priorities (≤12 months), medium-term priorities (1–3 years) and long-term priorities (3–5 years), distinguishing those led primarily by national governments from those requiring partner support or joint implementation (Table 3). This prioritisation highlights the need for immediate system strengthening actions to stabilise routine immunization performance, while simultaneously investing in medium- and long-term structural reforms to improve resilience, sustainability, and equity toward IA2030 targets.

First, as an immediate priority, immunization should be rebuilt from the ground up, starting with a return to immunization fundamentals, which is urgently needed through a revitalised Reach Every District (RED) strategy, supported by granular subnational analysis using tools such as the WHO Immunization League Table to identify and reach every zero-dose and incompletely vaccinated child. Catch-up vaccination must be institutionalised as a routine as part of EPI programme policies. Crucially, immunization should be fully integrated into primary health care and serve as an entry point for health system strengthening rather than being delivered as a vertical programme. Communities and civil society could be engaged at the core of planning, implementation, monitoring, and evaluation.

Second, over the short to medium term, immunization sovereignty could be achieved through sustainable financing and African innovation, including local manufacturing of vaccines. The fiscal realities of Gavi 6.0 demand a decisive strategic pivot. Rigorous vaccine portfolio prioritisation and optimisation through VPOP exercises is essential, enabling evidence-based choices. Governments should aggressively mobilise and secure domestic financing, honouring the Abuja, Addis Ababa, and Lusaka Declaration commitments. Accelerated investment in African vaccine manufacturing and joint or pooled vaccine procurement mechanisms is critical to build sub-regional sovereignty and resilience. These efforts would be complemented by adapted climate-resilient cold-chain and delivery networks, integrating climate and insecurity risk assessments into supply chain design, promoting vaccine technologies (heat stable, Patch, etc.) that reduce dependency on cold-chain and trained health workers. These will particularly be helpful to safeguard immunization services for continuity in conflict-affected, hard-to-reach and flood-prone areas.

Third, across both short- and medium-term horizons, the health workforce, which is the backbone of every health and immunization programme, should be prioritised. West African countries are encouraged to improve the number of health workers, their motivation, equitable distribution, and support, particularly at the community level. No strategy could succeed without skilled, supported, and empowered health workers reaching every child. Beyond relying solely on health workers, immunization programmes should serve as an entry point to institutionalise and strengthen a network of formal Community Health Workers, with appropriate micro-incentives within broader primary health care. These community-based actors, selected or formally recognised by the communities, would play a key role in building trust, supporting the identification and follow-up of zero-dose and under-immunized children, and addressing misinformation.

Fourth, as a medium- and long-term structural reform, outbreak prevention needs to be fundamentally reshaped. Existing fragmented VPD surveillance systems require a shift to integration, dissolving artificial silos between disease-specific surveillance and integrated disease surveillance. Governments are encouraged to domesticate surveillance financing in national plans and National Immunization Strategies, ending the perilous external dependency on volatile donor funds. WHO AFRO and partners are called to pioneer “Outbreak Risk Predictive Models”, leveraging machine learning to forecast risks months in advance. Such predictive capability would shift the paradigm from costly reactive campaigns toward targeted, preventive and risk reduction interventions that are most effective, efficient, and sustainable towards achieving IA30 targets.

Finally, mutual accountability should be ensured at different levels of national health systems and with partners. Robust monitoring of these engagements is essential, with quarterly reviews and a live dashboard tracking progress across all countries.

Taken together, this phased approach underscores the importance of immediate system stabilisation, followed by medium-term structural reforms to build resilient, equitable, and sustainable immunization systems anchored within primary health care in a robust health system aligned with IA2030.

Looking forward, progress toward IA2030 will require increased and sustained domestic financing of immunization, deliberate integration of vaccination within primary health care systems, and strengthened health workforce management including at community level. This would also require innovation to address climate, security, and access constraints. Future efforts should focus on translating these system-level actions into measurable national implementation plans and monitoring their impact on equity and coverage outcomes.

## 8. Conclusions

The 2025 West Africa EPI Managers’ Meeting occurred at an inflection point: either continue with incremental improvements to fragmented systems or embrace transformative change. EPI managers’ analyses suggest that the sub-region’s immunization challenges are systemic, interconnected with the overall health system challenges.

The health system framework shows a pathway from diagnosis to prescription. By linking problems to system failures, we move beyond the cycle of identifying familiar challenges within EPI programmes toward actually resolving them holistically. The call for action proposes immediate and mid-term actions that countries can implement with existing resources.

Ultimately, achieving IA2030 goals in West Africa requires acknowledging that technical solutions alone are insufficient. The subregion needs to revamp political commitment to sustainable financing and work towards the transformation of data systems, increased genuine community partnership in programme design, and greater investment in risk reduction interventions to prevent VPD outbreaks.

## Figures and Tables

**Table 1:** DTP1 and 3 and MCV1 and 2 coverage in West African countries, 2019–2024.

Country	DPT1						DPT3						MCV1						MCV2					
	2019	2020	2021	2022	2023	2024	2019	2020	2021	2022	2023	2024	2019	2020	2021	2022	2023	2024	2019	2020	2021	2022	2023	2024
Cabo Verde	96	93	93	93	93	93	96	93	93	93	93	93	98	95	95	95	95	95	91	86	86	86	86	86
Ghana	97	94	99	99	99	99	97	94	98	99	95	95	92	88	94	95	90	90	83	79	83	84	79	79
Sierra Leone	95	93	94	92	92	92	95	91	92	91	91	91	93	87	87	90	90	90	72	67	67	73	73	73
Burkina Faso	95	95	95	95	95	95	91	91	91	91	91	91	88	88	88	88	88	88	71	71	71	66	65	65
Algeria	95	94	92	91	98	98	88	84	81	77	92	92	80	80	79	79	99	94	76	74	73	71	93	90
Senegal	93	89	91	93	92	96	91	83	85	93	92	91	86	82	88	87	87	87	63	63	64	69	74	79
Mauritania	88	92	87	96	96	95	80	81	77	85	90	86	75	82	75	84	92	93					24	59
Togo	92	88	90	90	95	95	86	85	87	86	90	90	78	75	77	79	81	81	59	55	59	66	67	69
Gambia	93	88	82	85	84	82	88	85	82	79	84	80	85	82	79	74	80	83	61	61	61	52	73	83
Niger	92	93	94	96	94	95	81	81	82	84	85	86	79	79	80	65	80	81	58	60	66	42	68	77
Liberia	86	82	81	93	91	91	71	66	67	79	83	83	68	61	58	79	82	82	13	30	35	59	60	60
Mali	81	75	79	84	85	91	77	70	77	75	75	82	71	62	72	69	68	72	4	26	33	36	60	60
Guinea-Bissau	86	87	81	81	80	74	78	74	67	74	74	68	79	72	63	75	72	65				1	36	45
Cote d’Ivoire	87	77	78	78	83	80	79	72	73	73	79	77	71	63	65	62	70	75			1	20	31	48
Benin	81	80	80	79	79	76	72	70	70	69	69	63	58	54	53	52	52	44						
Nigeria	72	70	67	63	71	71	66	62	61	59	67	67	58	60	56	51	54	57	9	38	34	30	33	35
Guinea	67	68	70	71	73	77	53	54	56	58	60	63	50	50	51	51	52	60				3	26	45

**Table 2:** Progress toward IA2030 milestones in West Africa, 2025.

IA2030 Target	Status in West Africa	Assessment/Progress
▪ 90% coverage for all vaccines in all districts	Regional D PT3 median: 72%; 98 districts < 50% coverage (84% increased)	Off-track
▪ Reduce zero-dose children by 50%	Nigeria alone accounts for 31% of AFRO and 75% of West Africa’s zero-dose children; BCU only reached 53% of ZD, progress is highly variable among countries	Off-track
▪ Eliminate measles and rubella	Measles outbreaks expanded 34% (2023–2025); new districts affected	Off-track
▪ MNT elimination	16/17 countries (94.1%) already validated	On track
▪ Reduce VPD mortality by 50%	Stagnating coverage and recurrent outbreaks suggest a negative trajectory	Uncertain

**Table 3:** Priority matrix for implementation of strategic actions of EPICON25 in West Africa, 2025.

Timeframe	Government-Led Priorities	Partner Supported/Joint Priorities
Short-term (≤12 months)	Improve data quality and use for micro-planning (league table, triangulation) Revitalize Reach Every District (RED) strategy and integrate immunization within PHC Institutionalize big catch-up vaccination within routine services Strengthen subnational accountability through regular performance reviews Increase domestic funding for RI & Surveillance	Targeted technical assistance for data systems and monitoring, High Level Advocacy to Governments using regional & subregional bodies Rapid support to protect routine immunization during humanitarian shocks & outbreaks
Medium-term (1–3 years)	Sustain domestic financing Increase for routine immunization and vaccines Strengthen health workforce capacity and community-based delivery mechanisms Integrate immunization and VPD surveillance systems Local Vaccine Manufacturing & climate resilient adapted cold chain & deliveries	Support local vaccine manufacturing and pooled procurement mechanisms Invest in climate-resilient cold chain and delivery systems Develop outbreak-risk prediction and early-warning tools

## Data Availability

The data sources are available from the corresponding authors upon request from the Editor-in-Chief. WHO-UNICEF Estimates of National Immunization Coverage Estimates 2024 revision released 15 July 2025 (available at https://immunizationdata.who.int/, accessed on 14 April 2026) and eJRF accessible from https://www.who.int/data/immunization (accessed on 16 April 2026).

## Abbreviations

WHO	World Health Organization
WAHO	West African Health Organization
AMP	Agence de Médecine Préventive
UNICEF	United Nations Children’s Fund
GAVI	The Vaccine Alliance
PATH	Programme for Appropriate Technology in Health
GaneshAID	Women-led, humanist consultancy firm dedicated to improving global health, strengthening health systems, and accelerating health equity in low- and middle-income countries.
